# Crystal structure of bis­(3,5-di­methyl­pyridine-κ*N*)bis­(methanol-κ*O*)bis­(thio­cyanato-κ*N*)cobalt(II)

**DOI:** 10.1107/S2056989016018326

**Published:** 2016-11-18

**Authors:** Stefan Suckert, Inke Jess, Christian Näther

**Affiliations:** a Institut für Anorganische Chemie, Christian-Albrechts-Universität Kiel, Max-Eyth Strasse 2, D-24118 Kiel, Germany

**Keywords:** crystal structure, cobalt(II) thio­cyanate complex, 3,5-di­methyl­pyridine ligand, hydrogen bonding

## Abstract

The asymmetric unit of the title complex, [Co(NCS)_2_(C_7_H_9_N)_2_(CH_3_OH)_2_], comprises of one Co^II^ cation located on a centre of inversion, one thio­cyanate ligand, one methanol ligand and one 3,5-di­methyl­pyridine ligand. The Co^II^ cation is octa­hedrally coordinated by two terminal N-bonding thio­cyanate anions, two methanol mol­ecules and two 3,5-di­methyl­pyridine ligands into a discrete complex. The complex mol­ecules are linked by inter­molecular O—H⋯S hydrogen bonding into chains that elongate in the direction parallel to the *b* axis.

## Abstract 2

The crystal structure of the title cobalt(II) compound consists of discrete octa­hedral complexes that are linked by inter­molecular O—H⋯S hydrogen bonding into chains.

## Chemical context   

For a long time, the synthesis of new mol­ecular magnetic materials with desired physical properties has been a topic of inter­est in coordination chemistry (Liu *et al.*, 2015 ▸). To reach this goal, paramagnetic cations must be linked by small anionic ligands such as, for example, thio­cyanate anions that can mediate magnetic exchange between the cations (Palion-Gazda *et al.*, 2015 ▸; Massoud *et al.*, 2013 ▸). In this context, our group has already reported several thio­cyanato coordination polymers which – depending on the metal cation and the neutral co-ligand – show different magnetic phenomena including a slow relaxation of the magnetization (Werner *et al.*, 2014 ▸, 2015*a* ▸,*b* ▸,*c* ▸). In this regard, discrete complexes are also of inter­est because a transformation into the desired polymeric compounds can be achieved through thermal decomposition, as shown in one of our previous studies (Näther *et al.*, 2013 ▸). During our systematic work, compounds based on 3,5-di­methyl­pyridine as co-ligand should be prepared, for which only one thio­cyanato compound is known (Price & Stone, 1984 ▸; Nassimbeni *et al.*, 1986 ▸). In the course of our investigations with Co^II^ as the transition metal, crystals of the title compound, [Co(NCS)_2_(C_7_H_9_N)_2_(CH_3_OH)_2_], were obtained and characterized by single crystal X-ray diffraction. Unfortunately, no single-phase crystalline powder could be synthesized, which prevented further investigations of physical properties.

## Structural commentary   

**Figure 1:**
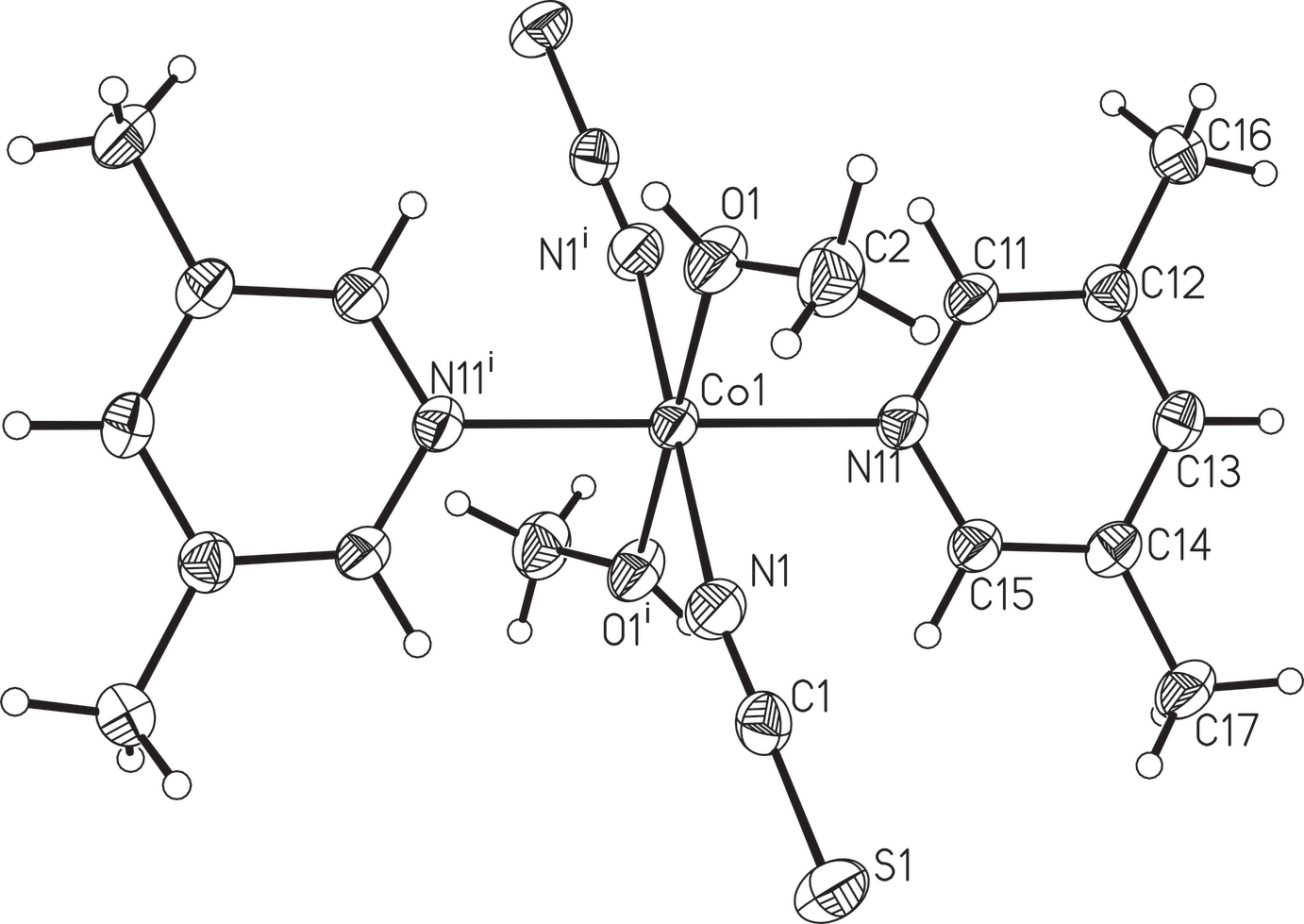
View of a discrete complex of the title compound, showing the atom labelling and anisotropic displacement ellipsoids drawn at the 50% probability level. [Symmetry code: (i) −*x*, −*y* + 1, −*z* + 1.]

The asymmetric unit of the title compound comprises of one Co^II^ cation, one thio­cyanato anion, one methanol mol­ecule and one neutral 3,5-di­methyl­pyridine co-ligand. The Co^II^ cation is located on a center of inversion; the thio­cyanate anion, the methanol mol­ecule as well as the 3,5-di­methyl­pyridine ligand are each located on general positions. The Co^II^ cation is octa­hedrally coordinated by two terminal N-bonded thio­cyanato ligands, two methanol mol­ecules and two 3,5-di­methyl­pyridine ligands in an all-*trans* configuration (Fig. 1 ▸). The Co—N bond length to the thio­cyanate anion is significantly shorter [2.0898 (19) Å] than to the pyridine N atom of the 3,5-di­methyl­pyridine ligand [2.1602 (17) Å], which is in agreement with values reported in the literature (Goodgame *et al.*, 2003 ▸; Wöhlert *et al.*, 2014 ▸).
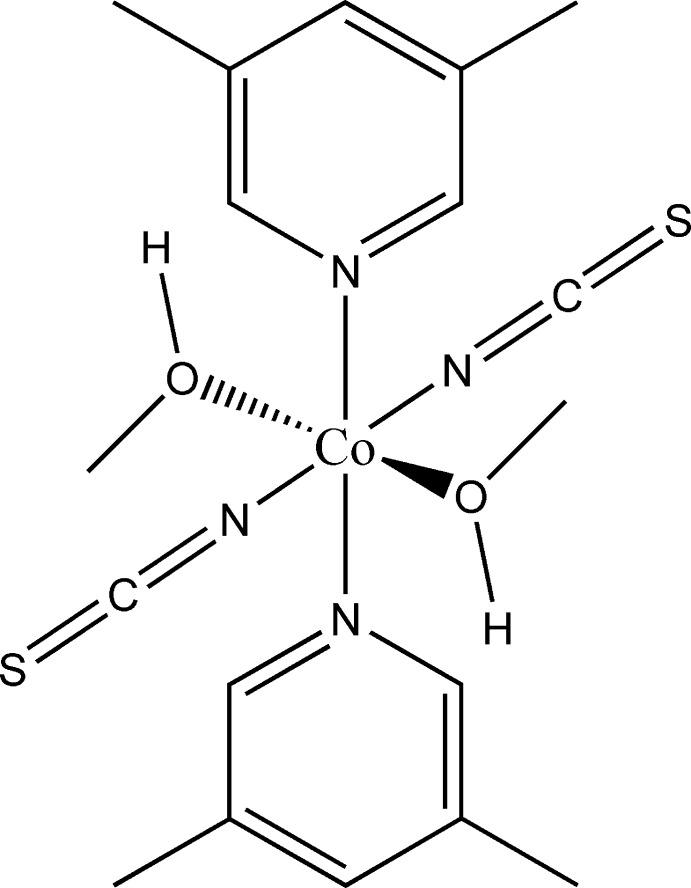


## Supra­molecular features   

**Figure 2:**
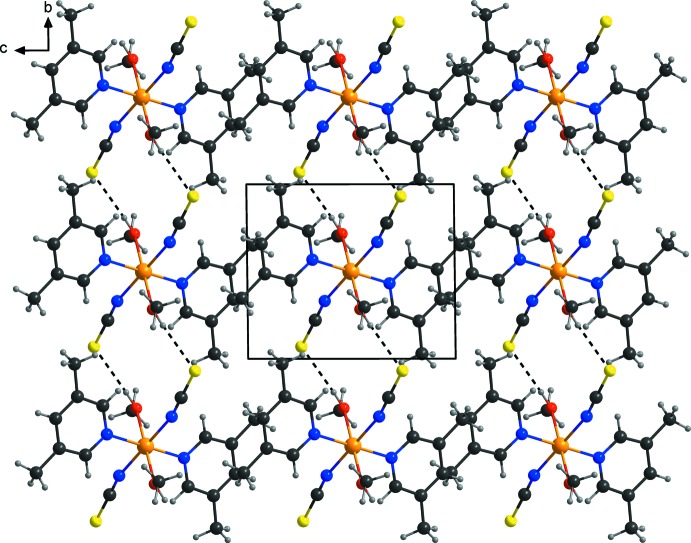
The crystal structure of the title compound in a view along the *a* axis, showing the inter­molecular hydrogen bonding as dashed lines.

The discrete complexes in the crystal are linked by pairs of inter­molecular O—H⋯S hydrogen bonds between the hydroxyl H atom of the methanol ligand and the thio­cyanato S atom of an adjacent complex into chains propagating parallel to the *b* axis (Fig. 2 ▸, Table 1 ▸). These pairs are located around centres of inversion.

## Database survey   

To the best of our knowledge, there is only one thio­cyanato coordination compound with 3,5-di­methyl­pyridine as a co-ligand deposited in the Cambridge Structure Database (Version 5.37, last update 2015; Groom *et al.*, 2016 ▸). The structure consists of an Ni^II^ cation octa­hedrally coordinated by four 3,5-di­methyl­pyridine ligands and two N-bonded thio­cyanate anions (Price *et al.*, 1984 ▸; Nassimbeni *et al.*, 1986 ▸). A general search for coordination compounds with 3,5-di­methyl­pyridine resulted in 159 structures, including the aforementioned ones. Exemplary are two Co compounds: in the first, the cation is octa­hedrally coordinated by two 3,5-di­methyl­pyridine ligands as well as one *μ*-1,3-bridging and one *μ*-1,1-bridging azide anion, linking them into chains (Lu *et al.*, 2012 ▸), whereas in the second compound, the Co^II^ atom is octa­hedrally coordinated by four 3,5-di­methyl­pyridine ligands and two chloride anions, forming a discrete complex (Martone *et al.*, 2007 ▸).

## Synthesis and crystallization   

Co(NCS)_2_ and 3,5-di­methyl­pyridine were purchased from Alfa Aesar. Crystals of the title compound suitable for single crystal X-ray diffraction were obtained by the reaction of 43.8 mg Co(NCS)_2_ (0.25 mmol) with 28.5 µl 3,5-di­methyl­pyridine (0.6 mmol) in methanol (1.5 ml) after a few days.

## Refinement   

Crystal data, data collection and structure refinement details are summarized in Table 2 ▸. The C—H hydrogen atoms were positioned with idealized geometry and were refined with fixed isotropic displacement parameters *U*_iso_(H) = 1.2*U*_eq_(C) using a riding model. The O—H hydrogen atom was located in a difference map. For refinement, the bond length was constrained to 0.84 Å, with *U*_iso_(H) = 1.5*U*_eq_(O), using a riding model.

## Supplementary Material

Crystal structure: contains datablock(s) I. DOI: 10.1107/S2056989016018326/wm5338sup1.cif

Structure factors: contains datablock(s) I. DOI: 10.1107/S2056989016018326/wm5338Isup2.hkl

CCDC reference: 1517370

Additional supporting information: crystallographic information; 3D view; checkCIF report

## Figures and Tables

**Table 1:** Hydrogen-bond geometry (Å, °)

*D*—H⋯*A*	*D*—H	H⋯*A*	*D*⋯*A*	*D*—H⋯*A*
O1—H1⋯S1^i^	0.84	2.45	3.2885 (17)	175

Symmetry code: (i) 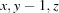.

**Table 2:** Experimental details

Crystal data	
Chemical formula	[Co(NCS)_2_(C_7_H_9_N)_2_(CH_4_O)_2_]
*M*_r_	453.48
Crystal system, space group	Triclinic, *P* 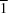
Temperature (K)	170
*a*, *b*, *c* (Å)	7.7027 (5), 7.8688 (5), 9.1970 (5)
α, β, γ (°)	87.403 (5), 81.419 (5), 76.295 (5)
*V* (Å^3^)	535.48 (6)
*Z*	1
Radiation type	Mo *K*α
μ (mm^−1^)	1.02
Crystal size (mm)	0.15 × 0.09 × 0.04

Data collection	
Diffractometer	Stoe IPDS2
Absorption correction	Numerical (*X-SHAPE* and *X-RED32*; Stoe & Cie, 2008 ▸)
*T*_min_, *T*_max_	0.885, 0.923
No. of measured, independent and observed [*I* > 2σ(*I*)] reflections	6258, 2431, 2052
*R*_int_	0.024
(sin θ/λ)_max_ (Å^−1^)	0.648

Refinement	
*R*[*F* ^2^ > 2σ(*F* ^2^)], *wR*(*F* ^2^), *S*	0.034, 0.094, 1.08
No. of reflections	2431
No. of parameters	127
H-atom treatment	H-atom parameters constrained
Δρ_max_, Δρ_min_ (e Å^−3^)	0.37, −0.37

Computer programs: *X-AREA* (Stoe & Cie, 2008 ▸), *SHELXS97* (Sheldrick, 2008 ▸), *SHELXL2014* (Sheldrick, 2015 ▸), *XP* in *SHELXTL* (Sheldrick, 2008 ▸), *DIAMOND* (Brandenburg, 1999 ▸) and *publCIF* (Westrip, 2010 ▸).

## supplementary crystallographic information

### Crystal data

**Table:**

[Co(NCS)_2_(C_7_H_9_N)_2_(CH_4_O)_2_]	*Z* = 1
*M**_r_* = 453.48	*F*(000) = 237
Triclinic, *P*1	*D*_x_ = 1.406 Mg m^−^^3^
*a* = 7.7027 (5) Å	Mo *K*α radiation, λ = 0.71073 Å
*b* = 7.8688 (5) Å	Cell parameters from 6258 reflections
*c* = 9.1970 (5) Å	θ = 2.2–27.4°
α = 87.403 (5)°	µ = 1.02 mm^−^^1^
β = 81.419 (5)°	*T* = 170 K
γ = 76.295 (5)°	Block, blue
*V* = 535.48 (6) Å^3^	0.15 × 0.09 × 0.04 mm

### Data collection

**Table:**

Stoe IPDS-2 diffractometer	2052 reflections with *I* > 2σ(*I*)
ω scans	*R*_int_ = 0.024
Absorption correction: numerical (X-SHAPE and X-RED32; Stoe & Cie, 2008)	θ_max_ = 27.4°, θ_min_ = 2.2°
*T*_min_ = 0.885, *T*_max_ = 0.923	*h* = −9→9
6258 measured reflections	*k* = −10→10
2431 independent reflections	*l* = −11→11

### Refinement

**Table:**

Refinement on *F*^2^	0 restraints
Least-squares matrix: full	Hydrogen site location: mixed
*R*[*F*^2^ > 2σ(*F*^2^)] = 0.034	H-atom parameters constrained
*wR*(*F*^2^) = 0.094	*w* = 1/[σ^2^(*F*_o_^2^) + (0.0503*P*)^2^ + 0.2412*P*] where *P* = (*F*_o_^2^ + 2*F*_c_^2^)/3
*S* = 1.08	(Δ/σ)_max_ = 0.001
2431 reflections	Δρ_max_ = 0.37 e Å^−^^3^
127 parameters	Δρ_min_ = −0.37 e Å^−^^3^

### Special details

**Table:**

Geometry. All esds (except the esd in the dihedral angle between two l.s. planes) are estimated using the full covariance matrix. The cell esds are taken into account individually in the estimation of esds in distances, angles and torsion angles; correlations between esds in cell parameters are only used when they are defined by crystal symmetry. An approximate (isotropic) treatment of cell esds is used for estimating esds involving l.s. planes.

### Fractional atomic coordinates and isotropic or equivalent isotropic displacement parameters (Å^2^)

**Table:**

	*x*	*y*	*z*	*U*_iso_*/*U*_eq_	
Co1	0.0000	0.5000	0.5000	0.02492 (13)	
N1	0.1343 (3)	0.6688 (3)	0.3719 (2)	0.0319 (4)	
C1	0.2064 (3)	0.7782 (3)	0.3253 (2)	0.0282 (4)	
S1	0.30808 (9)	0.93060 (7)	0.25699 (6)	0.03616 (16)	
O1	0.2263 (2)	0.2834 (2)	0.45808 (19)	0.0347 (4)	
H1	0.2416	0.1917	0.4098	0.042*	
C2	0.4115 (3)	0.2961 (4)	0.4380 (3)	0.0406 (6)	
H2A	0.4455	0.3340	0.3370	0.061*	
H2B	0.4891	0.1816	0.4566	0.061*	
H2C	0.4261	0.3815	0.5069	0.061*	
N11	0.1118 (3)	0.5678 (2)	0.68521 (19)	0.0268 (4)	
C11	0.1607 (3)	0.4492 (3)	0.7899 (2)	0.0281 (4)	
H11	0.1514	0.3327	0.7775	0.034*	
C12	0.2242 (3)	0.4871 (3)	0.9156 (2)	0.0282 (4)	
C13	0.2358 (3)	0.6584 (3)	0.9313 (2)	0.0291 (4)	
H13	0.2780	0.6900	1.0158	0.035*	
C14	0.1865 (3)	0.7841 (3)	0.8251 (2)	0.0287 (4)	
C15	0.1263 (3)	0.7319 (3)	0.7033 (2)	0.0272 (4)	
H15	0.0937	0.8165	0.6291	0.033*	
C16	0.2780 (3)	0.3476 (3)	1.0281 (2)	0.0343 (5)	
H16A	0.3882	0.2640	0.9867	0.051*	
H16B	0.1807	0.2864	1.0554	0.051*	
H16C	0.3001	0.4014	1.1155	0.051*	
C17	0.1983 (4)	0.9710 (3)	0.8375 (3)	0.0374 (5)	
H17A	0.2179	1.0218	0.7388	0.056*	
H17B	0.2991	0.9747	0.8899	0.056*	
H17C	0.0855	1.0382	0.8917	0.056*	

### Atomic displacement parameters (Å^2^)

**Table:**

	*U*^11^	*U*^22^	*U*^33^	*U*^12^	*U*^13^	*U*^23^
Co1	0.0324 (2)	0.0230 (2)	0.0224 (2)	−0.01104 (16)	−0.00600 (15)	−0.00096 (14)
N1	0.0419 (11)	0.0315 (9)	0.0264 (9)	−0.0160 (8)	−0.0056 (8)	0.0007 (7)
C1	0.0330 (11)	0.0299 (10)	0.0226 (9)	−0.0070 (9)	−0.0062 (8)	−0.0034 (8)
S1	0.0456 (4)	0.0304 (3)	0.0358 (3)	−0.0184 (3)	−0.0004 (3)	−0.0018 (2)
O1	0.0328 (9)	0.0303 (8)	0.0428 (9)	−0.0089 (7)	−0.0054 (7)	−0.0103 (7)
C2	0.0308 (12)	0.0427 (13)	0.0512 (14)	−0.0114 (10)	−0.0092 (10)	−0.0056 (11)
N11	0.0334 (10)	0.0255 (9)	0.0240 (8)	−0.0101 (7)	−0.0064 (7)	−0.0018 (7)
C11	0.0367 (12)	0.0226 (10)	0.0278 (10)	−0.0104 (9)	−0.0071 (8)	−0.0014 (8)
C12	0.0304 (11)	0.0300 (11)	0.0252 (10)	−0.0089 (9)	−0.0037 (8)	−0.0014 (8)
C13	0.0320 (11)	0.0314 (11)	0.0261 (10)	−0.0098 (9)	−0.0051 (8)	−0.0059 (8)
C14	0.0322 (11)	0.0260 (10)	0.0292 (10)	−0.0096 (9)	−0.0027 (8)	−0.0045 (8)
C15	0.0323 (11)	0.0249 (10)	0.0261 (10)	−0.0091 (8)	−0.0051 (8)	−0.0008 (8)
C16	0.0418 (13)	0.0339 (12)	0.0299 (11)	−0.0110 (10)	−0.0114 (9)	0.0027 (9)
C17	0.0480 (14)	0.0283 (11)	0.0410 (13)	−0.0161 (10)	−0.0097 (11)	−0.0050 (9)

### Geometric parameters (Å, º)

**Table:**

Co1—N1	2.0898 (19)	C11—C12	1.390 (3)
Co1—N1^i^	2.0898 (19)	C11—H11	0.9500
Co1—O1^i^	2.1311 (16)	C12—C13	1.388 (3)
Co1—O1	2.1311 (16)	C12—C16	1.502 (3)
Co1—N11^i^	2.1602 (17)	C13—C14	1.386 (3)
Co1—N11	2.1602 (17)	C13—H13	0.9500
N1—C1	1.164 (3)	C14—C15	1.388 (3)
C1—S1	1.636 (2)	C14—C17	1.505 (3)
O1—C2	1.438 (3)	C15—H15	0.9500
O1—H1	0.8399	C16—H16A	0.9800
C2—H2A	0.9800	C16—H16B	0.9800
C2—H2B	0.9800	C16—H16C	0.9800
C2—H2C	0.9800	C17—H17A	0.9800
N11—C11	1.342 (3)	C17—H17B	0.9800
N11—C15	1.342 (3)	C17—H17C	0.9800
			
N1—Co1—N1^i^	180.0	C15—N11—Co1	121.16 (14)
N1—Co1—O1^i^	87.76 (7)	N11—C11—C12	123.78 (19)
N1^i^—Co1—O1^i^	92.24 (7)	N11—C11—H11	118.1
N1—Co1—O1	92.24 (7)	C12—C11—H11	118.1
N1^i^—Co1—O1	87.76 (7)	C13—C12—C11	116.89 (19)
O1^i^—Co1—O1	180.0	C13—C12—C16	122.06 (19)
N1—Co1—N11^i^	92.30 (7)	C11—C12—C16	121.05 (19)
N1^i^—Co1—N11^i^	87.70 (7)	C14—C13—C12	120.79 (19)
O1^i^—Co1—N11^i^	89.25 (6)	C14—C13—H13	119.6
O1—Co1—N11^i^	90.75 (6)	C12—C13—H13	119.6
N1—Co1—N11	87.70 (7)	C13—C14—C15	117.59 (19)
N1^i^—Co1—N11	92.30 (7)	C13—C14—C17	122.42 (19)
O1^i^—Co1—N11	90.75 (6)	C15—C14—C17	120.0 (2)
O1—Co1—N11	89.25 (6)	N11—C15—C14	123.23 (19)
N11^i^—Co1—N11	180.0	N11—C15—H15	118.4
C1—N1—Co1	167.28 (17)	C14—C15—H15	118.4
N1—C1—S1	179.04 (19)	C12—C16—H16A	109.5
C2—O1—Co1	124.49 (14)	C12—C16—H16B	109.5
C2—O1—H1	97.4	H16A—C16—H16B	109.5
Co1—O1—H1	131.5	C12—C16—H16C	109.5
O1—C2—H2A	109.5	H16A—C16—H16C	109.5
O1—C2—H2B	109.5	H16B—C16—H16C	109.5
H2A—C2—H2B	109.5	C14—C17—H17A	109.5
O1—C2—H2C	109.5	C14—C17—H17B	109.5
H2A—C2—H2C	109.5	H17A—C17—H17B	109.5
H2B—C2—H2C	109.5	C14—C17—H17C	109.5
C11—N11—C15	117.71 (17)	H17A—C17—H17C	109.5
C11—N11—Co1	121.05 (13)	H17B—C17—H17C	109.5

Symmetry code: (i) −*x*, −*y*+1, −*z*+1.

### Hydrogen-bond geometry (Å, º)

**Table:**

*D*—H···*A*	*D*—H	H···*A*	*D*···*A*	*D*—H···*A*
O1—H1···S1^ii^	0.84	2.45	3.2885 (17)	175

Symmetry code: (ii) *x*, *y*−1, *z*.
